# Supplementary material for: Validation of helical symmetry parameters in the EMDB
Source: Acta Crystallogr D Struct Biol. 2025 Sep 4;81(Pt 10):527–34. doi: 10.1107/S2059798325007260 (PMC12485488; doi:10.1107/S2059798325007260)
Supplement: Supplementary file 2 [file d-81-00527-sup2.pdf]

STRUCTURAL BIOLOGY

Volume 81 (2025)

Supporting information for article:

Validation of helical symmetry parameters in EMDB

Daoyi Li, María Muñoz Pérez, Xiaoqi Zhang, Jiaqing Li and Wen Jiang

In the HI3D_link column, clickable links are provided to show the HI3D lattice plot with both the original deposited helical parameters (red arrow) and the validated helical parameters (yellow arrow). If the arrow does not point to a lattice point, the corresponding helical parameters are incorrect. If the arrow points to a lattice point but not the one closest to the equator line and the center origin, the corresponding helical parameters are correct but represent partial helical symmetries.
